# Symptomatic Congenital Cytomegalovirus Infection in a Preterm Very-Low-Birth-Weight Infant Treated With Intravenous Ganciclovir Followed by Oral Valganciclovir

**DOI:** 10.7759/cureus.92581

**Published:** 2025-09-17

**Authors:** Hiroki Kita, Keisuke Maeda, Takayuki Nukada, Yuka Ikeda, Mitsukazu Mamada

**Affiliations:** 1 Department of Pediatrics, Japanese Red Cross Wakayama Medical Center, Wakayama, JPN

**Keywords:** congenital cytomegalovirus infection, ganciclovir, preterm neonate, valganciclovir, very low birth weight

## Abstract

Oral valganciclovir (VGCV) therapy has been shown to be effective against congenital cytomegalovirus (CMV) infection, with improvements in both auditory and neurological outcomes. However, therapeutic strategies remain undefined for cases involving preterm infants born before 34 weeks of gestation or those with a birth weight below 1200 g. We report a case of a male neonate born at 32 weeks and four days of gestation, with a birth weight of 1168 g, diagnosed with symptomatic congenital CMV infection. Diagnosis was based on positive CMV-DNA in urine and clinical findings, including ventriculomegaly and thrombocytopenia. Intravenous ganciclovir (GCV) was initiated, followed by a transition to oral VGCV. A marked reduction in CMV-DNA levels in both blood and urine was observed, along with recovery of platelet counts. Auditory evaluation revealed normal hearing. In severe cases of symptomatic congenital CMV infection in preterm very-low-birth-weight (VLBW) infants, initial intravenous GCV administration before oral VGCV may represent a viable treatment option. Furthermore, with appropriate monitoring for adverse events, antiviral therapy can be administered safely in this high-risk population.

## Introduction

Congenital cytomegalovirus (CMV) infection, with a prevalence of 0.31% among all live births, is one of the most common congenital infections; of these cases, 23.9% are symptomatic and frequently result in neurological sequelae [1]. For symptomatic congenital CMV infection, two antiviral agents are available: intravenous ganciclovir (GCV) and its oral prodrug, valganciclovir (VGCV). Oral VGCV has become the first-line treatment for congenital CMV infection, whereas intravenous GCV is preferred in cases where intestinal absorption is a concern or in severe cases [2]. In Japan, a protocol of six months of oral VGCV administration is currently recommended. However, the investigator-initiated clinical trial that established this protocol excluded preterm infants born before 32 weeks of gestation as well as low birth weight infants weighing less than 1800 g at the initiation of treatment [3]. Moreover, comparative efficacy between intravenous GCV and oral VGCV has not been evaluated in preterm infants born before 32 weeks of gestation or in very-low-birth-weight (VLBW) infants weighing less than 1200 g [4]. Therefore, treatment strategies for preterm and low birth weight infants remain to be clarified. We report a case of a preterm, VLBW infant with severe symptomatic congenital CMV infection, in whom intravenous GCV therapy was initiated in the early neonatal period, followed by a switch to oral VGCV after confirming treatment efficacy.

## Case presentation

Maternal history

The mother was a 29-year-old multiparous female with no notable personal or family medical history. At 31 weeks and four days of gestation, fetal growth restriction (FGR) was suspected, and she was referred to our institution. Fetal ultrasound revealed an estimated fetal weight of -2.7 SD, consistent with FGR, along with ventriculomegaly. Serological testing showed maternal positivity for both CMV-specific IgG and IgM antibodies, raising suspicion for intrauterine CMV infection. As fetal growth arrest persisted, an emergency cesarean section was performed at 32 weeks and four days of gestation.

Physical examination

The male infant was delivered with a birth weight of 1,168 g (-2.8 SD), length of 36.2 cm (-2.6 SD), head circumference of 27.0 cm (-1.5 SD), and chest circumference of 23.0 cm (-1.7 SD), consistent with small for gestational age (SGA). No other notable physical abnormalities were observed.

Laboratory and imaging findings

**Figure 1:**
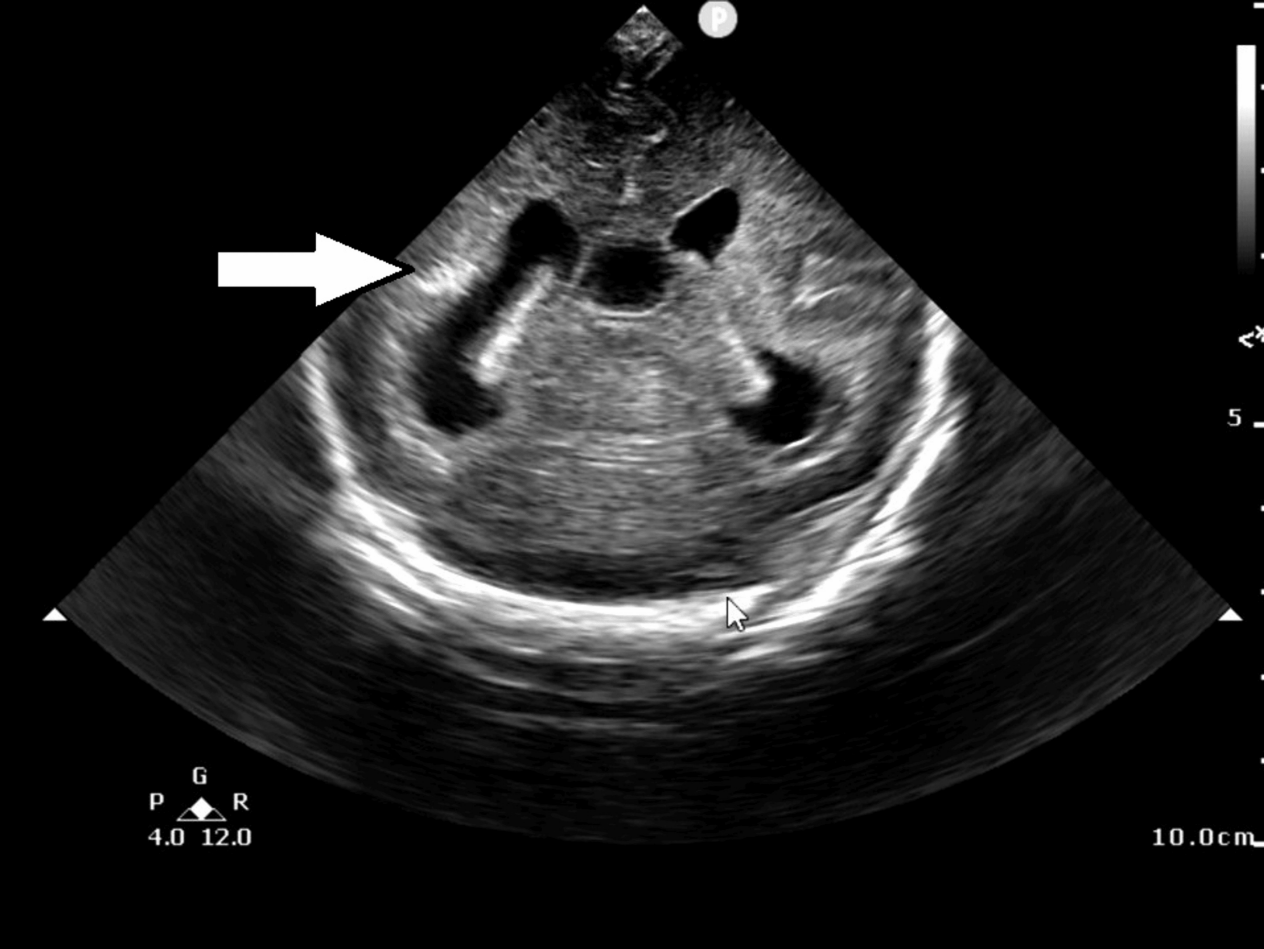
Ultrasound of the brain at birth (coronal view) Ventricular enlargement and intracranial calcification (white arrow) are observed

On day of life (DOL) 0, ultrasound of the brain revealed ventriculomegaly and intracranial calcifications (Figure 1).

On the same day, laboratory tests revealed decreased neutrophil and platelet counts (Table 1).

**Table 1:** Laboratory findings of DOL 0 WBC: white blood cell count; neutrophils: absolute neutrophil count; Hb: hemoglobin; PLT: platelet count; TP: total protein concentration; Alb: albumin concentration; ALP: alkaline phosphatase; AST: aspartate aminotransferase; ALT: alanine aminotransferase; LD: lactate dehydrogenase; BUN: blood urea nitrogen; Cre: creatinine; Na: sodium; K: potassium; Cl: chlorine; Ca: calcium; P: phosphorus; CRP: C-reactive protein

Investigation	Values	Reference range
WBC	3400	5000-19000 (/μL)
Neutrophils	850	2000-9000 (/μL)
Hb	14.9	16.9-21.1 (g/dL)
PLT	33000	80000-356000 (/μL)
TP	4.4	4.8-7.3 (g/dL)
Alb	3.1	4.1-5.1 (g/dL)
ALP	181	195-648 (U/L)
AST	51	11-59 (U/L)
ALT	14	4-21 (U/L)
LD	492	364-1120 (U/L)
BUN	8	4-20 (mg/dL)
Cre	0.55	0.3-0.9 (mg/dL)
Na	140	135-145 (mmol/L)
K	3.8	4.2-6.2 (mmol/L)
Cl	107	101-112 (mmol/L)
Ca	9.2	7.6-11.2 (mg/dL)
P	5.2	4.5-8.8 (mg/dL)
CRP	<0.01	0-0.14 (mg/dL)
IgG	345	831-1231 (mg/dL)
IgM	19	6-16 (mg/dL)

Fundoscopic examination on DOL 19 revealed CMV retinitis. A urine sample collected on DOL 1 tested positive for CMV by nucleic acid amplification testing, leading to a diagnosis of symptomatic congenital CMV infection. Quantitative CMV-DNA levels on DOL 14 were 1.8 × 10⁷ IU/mL in blood and 4.4 × 10⁸ IU/mL in urine. Placental histopathology demonstrated villitis with intranuclear inclusions, and CMV immunostaining was positive, confirming CMV-associated villitis.

**Figure 2:**
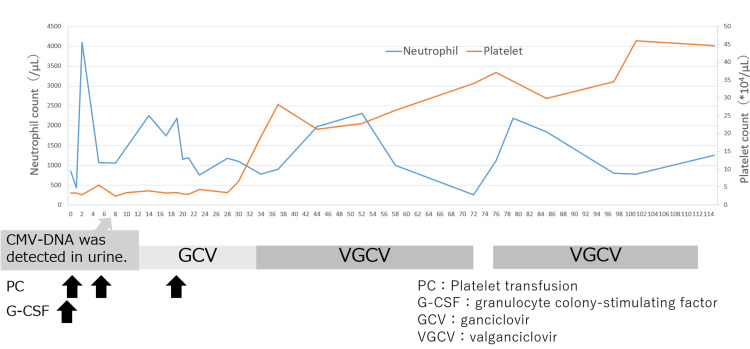
Clinical course and hematologic trends

**Figure 3:**
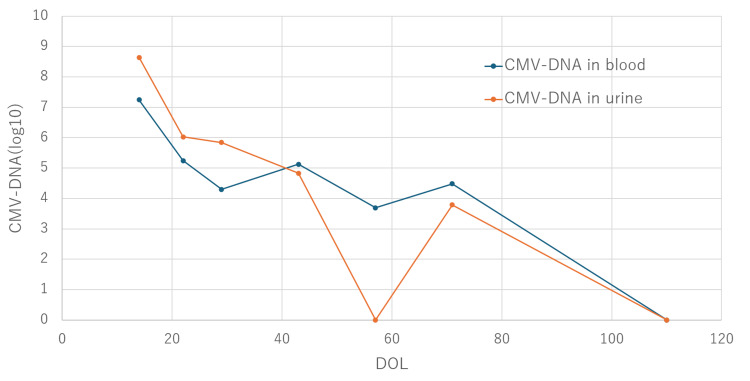
Trends in CMV-DNA levels in blood and urine CMV: cytomegalovirus

Clinical Course: At DOL 1, because the absolute neutrophil count fell below 500/μL, granulocyte colony-stimulating factor (G-CSF) was administered. In addition, platelet transfusions were performed as needed with a cutoff value of 30,000/μL, while the patient was closely monitored. Intravenous GCV at a dose of 6 mg/kg/dose twice daily was initiated on DOL 14. Before treatment, the patient required platelet transfusions due to thrombocytopenia; however, after initiation of intravenous GCV, platelet counts stabilized within the normal range, and transfusions were no longer required after DOL 20. Improvement in CMV retinitis was confirmed on DOL 33, and a decline in both blood and urinary CMV-DNA levels was observed. To monitor for neutropenia, blood counts were performed twice weekly, and absolute neutrophil counts remained above 500/μL. Upon confirming clinical improvement and viral load reduction, treatment was transitioned on DOL 34 to oral VGCV at 16 mg/kg/dose twice daily. Following the switch, CMV-DNA levels in both blood and urine continued to decline. On DOL 71, neutrophil counts dropped below 500/μL, necessitating temporary discontinuation of VGCV. Treatment was resumed on DOL 75 after recovery, and thereafter, neutrophil counts ranged within the normal range (Figures 2, 3).

**Figure 4:**
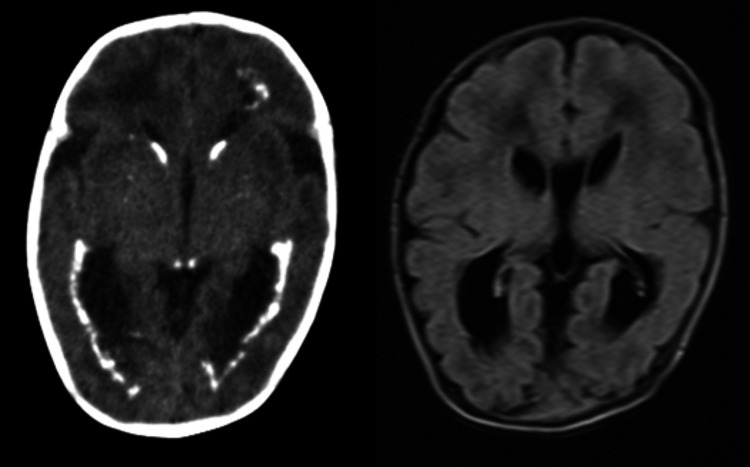
CT of the brain (left) and MRI (axial FLAIR Image, right) on day of life 51 CT: computed tomography; FLAIR: fluid-attenuated inversion recovery; MRI: magnetic resonance imaging

CT of the brain performed on DOL 51 (corrected age: 39 weeks and six days) showed bilateral ventriculomegaly and periventricular calcifications. MRI of the brain revealed delayed myelination and polymicrogyria involving the bilateral temporal, parietal, and occipital lobes (Figure 4).

At the time of this report, the patient was eight months of chronological age (six months corrected age). In terms of motor development, head control had been achieved; however, rolling over and independent sitting were not yet attained. In the language domain, babbling had not been acquired. These findings indicate developmental delay even when adjusted for corrected age. Auditory brainstem response (ABR) and distortion product otoacoustic emissions (DPOAE) performed on DOL 54 (corrected age: 40 weeks and two days), at four months (corrected age: two months), and at seven months (corrected age: five months) all demonstrated normal findings. Funduscopic examination at DOL 33 showed improvement of CMV retinitis, with no recurrence thereafter. Retinopathy of prematurity was present, and poor vascular extension was initially observed; however, the patient was followed up until eight months of age, during which spontaneous vascular extension occurred, and ophthalmologic follow-up was subsequently discontinued.

## Discussion

In this case of symptomatic congenital CMV infection in a preterm, VLBW infant, two key findings were demonstrated. First, in severe cases, intravenous GCV before oral VGCV may represent a viable treatment option. Second, short-term intravenous GCV can be safely administered with appropriate monitoring through regular hematologic testing. In symptomatic congenital CMV infection among severely affected preterm and VLBW infants, initiating treatment with intravenous GCV before oral VGCV may constitute a potential therapeutic approach. The clinical presentation of symptomatic CMV infection varies widely in severity. According to a review by Rawlinson et al., the presence of multiple findings-including central nervous system abnormalities, thrombocytopenia, petechiae, hepatosplenomegaly, and intrauterine growth restriction (IUGR) -supports a diagnosis of moderate to severe symptomatic CMV infection [5]. Multivariate analyses of prognostic factors have identified IUGR as an independent predictor of poor outcomes, including death and long-term care needs [6].

In the present case, the patient exhibited ventriculomegaly, intracranial calcifications, thrombocytopenia, and FGR, all indicative of a severe phenotype. Previous studies have demonstrated that intravenous GCV (6 mg/kg/dose twice daily for six weeks) improves hearing outcomes, prevents hearing deterioration, and supports neurodevelopment in symptomatic infants [7,8]. Kimberlin et al. further reported that the pharmacologic activity of intravenous GCV is equivalent to that of oral VGCV (16 mg/kg/dose twice daily) [4]. However, their study included only infants ≥32 weeks’ gestational age and ≥1200 g at treatment initiation; in fact, all analyzed cases were ≥34 weeks. Furthermore, the investigator-initiated clinical trial conducted in Japan did not include preterm infants born before 32 weeks of gestation or low birth weight infants weighing less than 1,800 g at the initiation of therapy [3].

It remains inconclusive whether treatment for symptomatic congenital CMV infection in preterm and low birth weight infants should be initiated with intravenous GCV or oral VGCV. Real-world data from the United States suggest that it is common to administer intravenous GCV before oral VGCV. In these data, the median age at VGCV initiation in the VGCV-only group was 45 days, which does not align with current treatment recommendations. Additionally, higher mortality rates in the GCV group imply that intravenous GCV is preferentially used in more severe cases [9]. Given the severe clinical findings in our case-including ventriculomegaly, intracranial calcifications, thrombocytopenia, and FGR-we opted to administer intravenous GCV before oral VGCV. This approach led to a reduction in CMV-DNA levels in blood and urine, improvement in thrombocytopenia, and resolution of retinitis. Accordingly, the patient was transitioned to oral VGCV after 20 days of intravenous GCV therapy. CMV-DNA levels continued to decline post-transition, with no recurrence of thrombocytopenia or retinitis. Although the patient demonstrated developmental delay at eight months of age, auditory assessments remained normal. We consider that the administration of intravenous GCV before oral VGCV may have contributed to this favorable auditory outcome.

Short-term intravenous GCV can be administered safely with regular hematologic monitoring. Neutropenia is the most common adverse event associated with GCV. Previous reports indicate that Grade 3-4 neutropenia occurred in 63% of infants treated with six weeks of intravenous GCV, 38% of those who received two weeks of GCV followed by four weeks of VGCV, and 19% of those who received six weeks of VGCV alone [10]. In the present case, 20 days of intravenous GCV were administered with biweekly blood tests, and no neutropenic events were observed during this period. Although neutropenia (absolute neutrophil count <500/μL) developed after transitioning to VGCV, the condition resolved promptly after treatment interruption and did not result in infectious complications. These findings suggest that with regular monitoring, early detection of adverse effects enables safe administration of antiviral therapy.

This case demonstrates that in symptomatic congenital CMV infection in severely affected preterm and VLBW infants, early intravenous GCV administration before oral VGCV may be a viable therapeutic option. While neutropenia is a known adverse event associated with GCV, our experience indicates that regular hematologic monitoring enables safe use. The optimal timing and sequencing of intravenous GCV and oral VGCV in this vulnerable population remain subjects for further investigation, and accumulation of additional clinical cases will be essential to establish evidence-based treatment strategies.

## Conclusions

GCV administered before VGCV may represent a viable therapeutic option for preterm and VLBW infants with severe congenital CMV infection. Careful monitoring for adverse events is essential to ensure treatment safety. Given that therapeutic strategies for congenital CMV infection in preterm and low birth weight infants remain undefined, further accumulation and analysis of clinical cases are required.

## References

[1] Ito Y, Morioka I, Takahashi N (2024). Clinical practice guidelines for the management of congenital cytomegalovirus infection in japan 2023: executive summary. Pediatr Infect Dis J.

[2] Szulc W, Szydłowska N, Smyk JM, Majewska A (2024). Progress and challenges in the management of congenital cytomegalovirus infection. Clin Pract.

[3] Morioka I, Kakei Y, Omori T (2022). Oral valganciclovir therapy in infants aged ≤2 months with congenital cytomegalovirus disease: a multicenter, single-arm, open-label clinical trial in Japan. J Clin Med.

[4] Kimberlin DW, Acosta EP, Sánchez PJ (2008). Pharmacokinetic and pharmacodynamic assessment of oral valganciclovir in the treatment of symptomatic congenital cytomegalovirus disease. J Infect Dis.

[5] Rawlinson WD, Boppana SB, Fowler KB (2017). Congenital cytomegalovirus infection in pregnancy and the neonate: consensus recommendations for prevention, diagnosis, and therapy. Lancet Infect Dis.

[6] Ito Y, Kimura H, Torii Y (2013). Risk factors for poor outcome in congenital cytomegalovirus infection and neonatal herpes on the basis of a nationwide survey in Japan. Pediatr Int.

[7] Kimberlin DW, Lin CY, Sánchez PJ (2003). Effect of ganciclovir therapy on hearing in symptomatic congenital cytomegalovirus disease involving the central nervous system: a randomized, controlled trial. J Pediatr.

[8] Oliver SE, Cloud GA, Sánchez PJ (2009). Neurodevelopmental outcomes following ganciclovir therapy in symptomatic congenital cytomegalovirus infections involving the central nervous system. J Clin Virol.

[9] Leung J, Grosse SD, Yockey B, Lanzieri TM (2022). Ganciclovir and valganciclovir use among infants with congenital cytomegalovirus: data from a multicenter electronic health record dataset in the United States. J Pediatric Infect Dis Soc.

[10] Kimberlin DW, Jester PM, Sánchez PJ (2015). Valganciclovir for symptomatic congenital cytomegalovirus disease. N Engl J Med.

